# The frequency of CD4+ and CD8+ circulating T stem cell memory in type 1 diabetes

**DOI:** 10.1002/iid3.715

**Published:** 2022-09-23

**Authors:** Pooriya Fazeli, Atefe Ghamar Talepoor, Zahra Faghih, Nasser Gholijani, Mohammad Reza Ataollahi, Mohammad Ali‐Hassanzadeh, Hossein Moravej, Kurosh Kalantar

**Affiliations:** ^1^ Department of Immunology School of Medicine Shiraz University of Medical Sciences Shiraz Iran; ^2^ Shiraz Institute for Cancer Research School of Medicine Shiraz University of Medical Sciences Shiraz Iran; ^3^ Autoimmune Diseases Research Center Shiraz University of Medical Sciences Shiraz Iran; ^4^ Department of Immunology School of Medicine Fasa University of Medical Sciences Fasa Iran; ^5^ Department of Immunology School of Medicine Jiroft University of Medical Sciences Jiroft Iran; ^6^ Department of Pediatrics School of Medicine Shiraz University of Medical Sciences Shiraz Iran

**Keywords:** autoimmunity, CD4 T cell, CD8 T cell, T memory stem cells, type 1 diabetes

## Abstract

**Introduction:**

The frequencies and functions of T stem cell memory (TSCM) subsets vary in autoimmune diseases. We evaluated the frequencies of CD4^+^ and CD8^+^ TSCM subsets as well as their PD‐1 expression levels in patients with T1D.

**Methods:**

Blood samples were collected from new case (NC) (*n* = 15), and long‐term (LT) (*n* = 15) groups and healthy controls (*n* = 15). Five subsets of T cells including TCM(CD4^+^/CD8^+^ CCR7^+^ CD45RO^+^ CD95^+^), TCM^hi^ (CD4^+^/CD8^+^ CCR7^+^ CD45RO^hi^ CD95^+^), TEM(CD4^+^/CD8^+^ CCR7^−^ CD45RO^+^ CD95^+^), TSCM(CD4^+^/CD8^+^ CCR7^+^ CD45RO^−^ CD95^+^), and T naive (CD4^+^/CD8^+^ CCR7^+^ CD45RO^−^ CD95^−^) were detected by flow‐cytometry.

**Results:**

The frequency of CD4^+^ TSCM was higher in NC patients than LT patients and controls (*p* < .0001 and *p* = .0086, respectively). A higher percentage of the CD8^+^ T naive cells was shown in NC patients as compared with LT and healthy individuals (*p* = .0003 and *p* = .0002, respectively). An increased level of PD‐1 expression was observed on the CD4^+^TCM and TCM^hi^ cells in LT patients as compared with healthy controls (*p* = .0037 and *p* = .0145, respectively). Also, the higher PD‐1 expression was observed on the CD8^+^ TCM and TCM^hi^ in NC and LT patients as compared with controls (*p* = .0068 and *p* < .0001; *p* = .0012 and *p* = .0012, respectively).

**Conclusion:**

Considering TSCMs' capacities to generate all memory and effector T cells, our results may suggest a potential association between the increased frequencies of TSCMs and T1D progression.

## Graphical abstract

In this study, we have checked the frequency of T stem cell memory (TSCM) in type 1 diabetes (T1D) and our results may suggest a potential association between the increased frequencies of TSCMs and T1D progression.

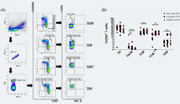

## 1 INTRODUCTION

Type 1 diabetes (T1D) is a chronic autoimmune disease resulting from the destruction of insulin‐producing β‐cells with strong genetic background and environmental triggers.^1^, ^2^ Insulitis, an inflammatory lesion of the islet due to β cell loss, is the pathologic hallmark of T1D.^2^ Different immune cells such as CD4^+^ and CD8^+^ T cells, B cells, dendritic cells (DC), and macrophages, as well as the production of islet‐specific autoantibodies, are involved in insulitis and T1D progression.^3^, ^4^, ^5^ The recognition of autoantigens like insulin, the 65‐kDa form of glutamic acid decarboxylase (GAD65), insulinoma‐associated protein 2 (IA‐2), and zinc transporter 8 (ZnT8) through the major histocompatibility complex (MHC), leads to T cell activation and contributes to disease development.^6^ Previous documents showed that GAD65 and (pro) insulin‐responsive T cells exhibited the properties of naive T cell compartment in healthy individuals, while in patients with T1D, GAD65 and (pro) insulin‐responsive T cells showed the characteristics of the naive and memory T cell compartments.^7^ Moreover, autoantigen‐responsive memory T cells in patients with T1D display a higher proliferative capacity to generate memory autoimmune T cell repertoire.^7^, ^8^ It has been shown that β‐cells‐responsive T cells found in T1D display features of antigen experience, expression of memory markers, decreased telomere length, and activation in the absence of costimulatory molecules.^7^, ^9^, ^10^ Furthermore, IL‐7 signaling plays an important role in the generation and maintenance of autoimmune T cells in T1D, as well as providing crucial signals for the generation of TSCM from naive precursors.^11^, ^12^ Additionally, the activation of autoimmune memory T cells was found in patients with T1D receiving pancreas and islet allografts 1 year after disease progression, which suggests the autoimmunity recurrence due to long‐lasting memory autoimmunity.^13^ The extent of T cell response depends on the activation of costimulatory or co‐inhibitory receptors after the engagement of T cell receptor (TCR) with the specific peptide‐MHC complex. Programmed cell death protein 1 (PD‐1), cytotoxic T‐lymphocyte‐associated protein 4 (CTLA‐4), lymphocyte‐activation gene 3 (LAG‐3), and T cell immunoglobulin and ITIM domain (TIGIT) are known as co‐inhibitory receptors.^14^ PD‐1 expression on T cells upon TCR stimulation induces anergy and exhaustion of T cells and is associated with persistent activation of self‐reactive T cells in autoimmune diseases. Certainly, depletion of PD‐1^+^ T cells has given beneficial effect in autoimmune disease through restriction of inflammation and disease progression.^15^, ^16^, ^17^

T1D pathogenesis involves the three main memory CD4^+^ and CD8^+^ T cell subsets, including central memory (CM; CD45RO^+^ CD45RA^−^ CCR7^+^ CD28^+^), transitional memory (TM; CD45RO^+^CD45RA^−^CCR7^−^ CD28^+^), and effector memory (EM; CD45RO^+^ CD45RA^−^ CCR7^−^CD28^−^ CD62L^low^).^18^ In addition to the previously known memory T cell subsets, a distinct subset of peripheral blood CD4^+^and CD8^+^ memory T cells, T stem cell memory (TSCM), has recently been discovered.^19^ TSCM is described as CD45RA^+^, CD45RO^−^, CCR7^+^, CD28^+^, CD95^+^, CD122^+^ (IL‐2Rβ), CD58^+^ and CD11a^+^ cell and comprises 2%–3% of memory T cells in humans.^20^, ^21^ Although TSCM preserves their naive‐like phenotype, they have self‐renewal capacity and are able to differentiate into CM and EM subsets.^22^, ^23^ The stem cell‐like functional properties of TSCM empower them to drive different diseases like aplastic anemia (AA), autoimmune uveitis,^24^ systemic lupus erythematosus (SLE),^25^ juvenile idiopathic arthritis or chronic lymphocytic leukemia (CML),^26^ immune thrombocytopenia (ITP),^27^ and HIV‐1 infection.^28^, ^29^ Previous studies have shown that TSCM can generate all memory and effector T cell subsets, which contribute to the progression of autoimmune diseases.^30^, ^31^, ^32^ In this context, these cells exhibit effector functions due to TNF‐α, IFN‐γ, and IL‐2 secretion.^33^ Therefore, imbalances in TSCM frequencies and functions can participate in the pathogenesis of different diseases. Collectively, the mentioned characteristics of the autoreactive T cell response in patients with T1D indicate that autoreactive TSCM can be generated during the pathogenesis of the disease. However, little is known about the distribution and function of different TSCM subsets in T1D.

In the present study, we investigated the frequency of CD4^+^ and CD8^+^ TSCM subsets as well as their PD‐1 expression levels in patients with T1D.

## 2 MATERIALS AND METHODS

### 2.1 Patients

A total of 15 new cases (NCs) with at least 1 year of T1D (5 females and 10 males, mean age ± SEM = 11.11 ± 1.36 years), 15 long‐term (LT) individuals with at least 5 years of T1D (3 females and 12 males, mean age ± SEM = 15.6 ± 1.34 years) and 15 healthy age and sex‐matched controls (six females and nine males, mean age ± SEM = 16.4 ± 1.72 years) were entered in this case‐control study. All subjects with T1D were selected from individuals referred to the Imam Reza clinic affiliated with Shiraz University of Medical Sciences over the past year. Demographic characteristics and laboratory data were collected during admission and are summarized in Table 1. The exclusion criteria included a positive history of alcohol, hepatic infections, HIV, cancers, autoimmune diseases, and pregnancy. The protocols were approved by the ethics committee of the medical school of Shiraz University of Medical Sciences (IR.sums.med.rec.1398.1107), and as the participants of the study were under 18 years old, the consent form was signed by their parents.

**Table 1:** The demographic and clinical characteristics of the study participants

Characteristic (mean ± SEM)	Patients		HC (*N* = 15)	*p*‐value
	NC (*N* = 15)	LT (*N* = 15)		
Age (years)	11.11 ± 1.36	15.6 ± 1.34	16.4 ± 1.72	.16
Gender (M/F)	10/5	12/3	9/6	.9
Diabetes duration (year)	1.5 ± 0.18	8.38 ± 0.88	_	<.0001
FBS (mg/dl)	261.64 ± 21.68	225.46 ± 24.48	_	.25
2hpp (mg/dl)	378.22 ± 61.94	221.45 ± 21.9	_	.021
HbA1C (%)	9.71 ± 0.39	9.19 ± 0.63	_	.44
Total daily insulin (U/kg/day)	27.13 ± 4.48	53 ± 7.81	_	.012
BMI (kg/m^2^)	16.75 ± 0.86	18.41 ± 0.94	_	.16
Plasma albumin (mmol/L)	9.55 ± 1.78	25.2 ± 12.6	_	.22
TG (mmol/L)	152.38 ± 48.4	101.43 ± 20.18	_	.77
HDL cholesterol (mmol/L)	48.57 ± 4.42	48.5 ± 3.99	_	.8
LDL cholesterol (mmol/L)	58.57 ± 11.6	104.5 ± 14.72	_	.34

Abbreviations: LT, long‐term; NC, new case.

### 2.2 Peripheral blood mononuclear cell (PBMC) isolation

Fresh heparinized blood samples (6 ml) were collected separately from each participant, and PBMCs were isolated by density‐gradient centrifugation at ×800*g* for 30 min at 25°C using Ficoll‐Paque Plus (inno‐Train Diagnostic GmbH). Then, freshly isolated PBMCs were resuspended to 1 × 10^6^ per ml in RPMI‐1640 culture medium (Shellmax), containing 10% fetal bovine serum and incubated overnight at 37°C.

### 2.3 Flow cytometry

PBMCs were stained at 4°C for 30 min with fluorochrome‐conjugated antibodies to characterize TSCM subsets. The following human conjugated monoclonal antibodies were used: anti‐CD4‐PerCP‐Cy5.5 (RPA‐T4), anti‐CD8‐APC‐Fire (RPA‐T8), anti‐CD45RO‐APC (UCHL1), anti‐CCR7FITC (G043H7), anti‐CD95‐PE (Dx2), and anti‐PD‐1‐PE‐Dazzle (EH12.2H7) from BioLegend. Mononuclear cells were separated from peripheral blood and live lymphocytes were identified by forward and side angle light scattering characteristics. Each T cell subset was defined as follows based on previous studies^24^, ^34^: T central memory (TCM): CD4 + (CD8) + CCR7 + CD45RO + CD95 + T cells; TCM^hi^: CD4 + (CD8) + CCR7 + CD45RO^hi^ CD95^+^ T cells; T effector memory (TEM): CD4^+^ (CD8)^+^ CCR7^−^ CD45RO^+^ CD95^+^ T cells; TSCM: CD4^+^ (CD8)^+^ CCR7^+^ CD45RO^−^ CD95^+^ T cells and T naive: CD4^+^ (CD8)^+^ CCR7^+^ CD45RO^−^ CD95^−^ T cells. At least 100,000 events per sample were collected using FACS Aria II (BD Sciences); and results were analyzed using FlowJo software (v9.6).

### 2.4 Statistical analysis

All statistical analyses were carried out using SPSS version 25 and Graphpad Prism version 8 software, and data were expressed as mean ± SEM. Normality was assessed by the Kolmogorov–Smirnov test. The student's *t*‐test or Mann–Whitney *U* test was used to evaluate the differences between two groups. The Kruskal–Wallis test was used for comparison of variables between more than two groups. The Spearman's rho method was used to evaluate the potential correlation between variables. *p* < .05 were considered statistically significant. The following symbols were applied to indicate statistically significant findings: *p < .05, **p < .01, ***p < .001, and ****p < .0001.

## 3 RESULTS

### 3.1 Demographic and clinical parameters

The demographic and clinical characteristics of patients with T1D and controls are demonstrated in Table 1. The duration of diabetes (*p* < .0001) and the total daily dose of insulin (*p* = .012) were significantly higher in patients with LT T1D compared to NC‐T1D individuals. Conversely, the 2hpp blood glucose level (*p* = .021) was higher in the NCs of T1D patients as compared with LT‐T1D individuals. Additionally, there were no significant differences in FBS, HbA1C, BMI, plasma albumin, TG, HDL, and LDL cholesterol between NC and LT T1D patients.

### 3.2 Frequency of CD4^+^ T cell subsets in T1D

**Figure 1:**
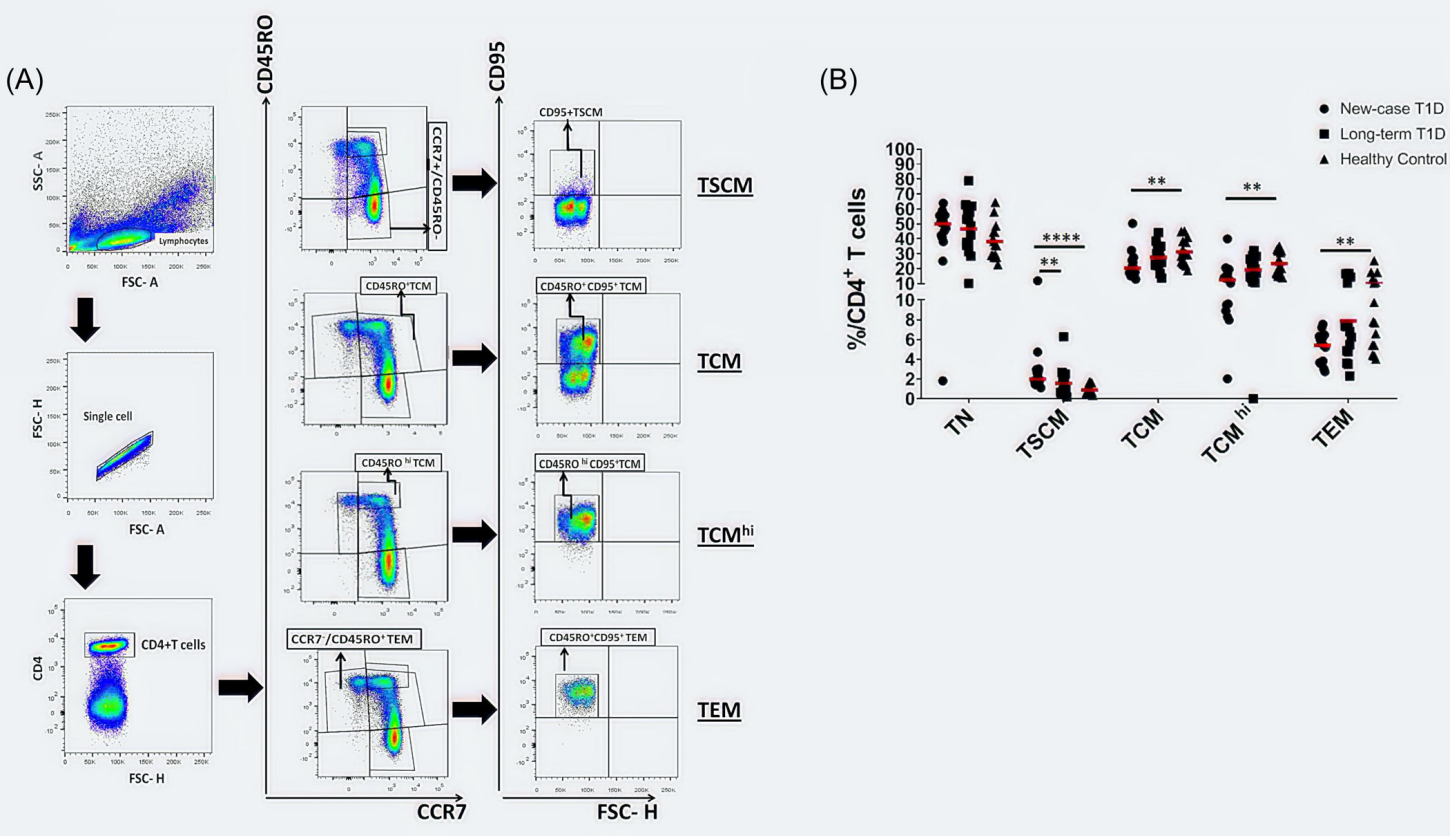
Changes in the percentages of CD4^+^T cell subsets in T1D patients. (A) The schematic diagram of gating of CD4^+^ T cell subsets in FCM. (B) The percentages of CD4^+^T cell subsets (TN, TSCM, TCM, TCM^hi^, and TEM) in new‐case T1D patients (*n* = 15), long‐term T1D patients (*n* = 15), and healthy controls (*n* = 15) are shown. Each symbol represents an individual. The means are represented by horizontal lines. *p < .05, **p < .01, ***p < .001, and ****p < .0001. Data analysis using the Kruskal–Wallis test followed by Bonferroni. TCM, T central memory.

All CD4^+^T cell subsets (TN, TSCM, TCM, TCM^hi^, and TEM) in the peripheral blood of all study subjects were identified by sequential surface marker gating as shown in Figure 1A. We found that the frequency of CD4^+^CCR7^+^CD45RO^−^CD95^+^ TSCM was higher in NC patients than LT patients and controls (2.87 ± 0.69 vs. 1.55 ± 0.38, *p* < .0001 and 2.87 ± 0.69 vs. 0.88 ± 0.12, *p* = .0086, respectively, Figure 1B). The percentage of CD4^+^CCR7^+^CD45RO^+^CD95^+^TCM was decreased in NC patients as compared with healthy controls (22.48 ± 2.4 vs. 31.23 ± 2.18, *p* = .0023, Figure 1B). As well, the frequency of CD4^+^CCR7^+^ CD45RO^hi^ CD95^+^TCM^hi^ was lower in NC patients than in healthy controls (14.21 ± 2.26 vs. 23.43 ± 1.94, *p* = .0014, Figure 1B). The percentage of CD4^+^CCR7^−^CD45RO^+^CD95^+^TEM was lower in NC patients than in healthy controls (5.08 ± 0.40 vs. 10.01 ± 1.57, *p* = .0033, Figure 1B).

### 3.3 Frequency of CD8^+^ T cell subsets in T1D

**Figure 2:**
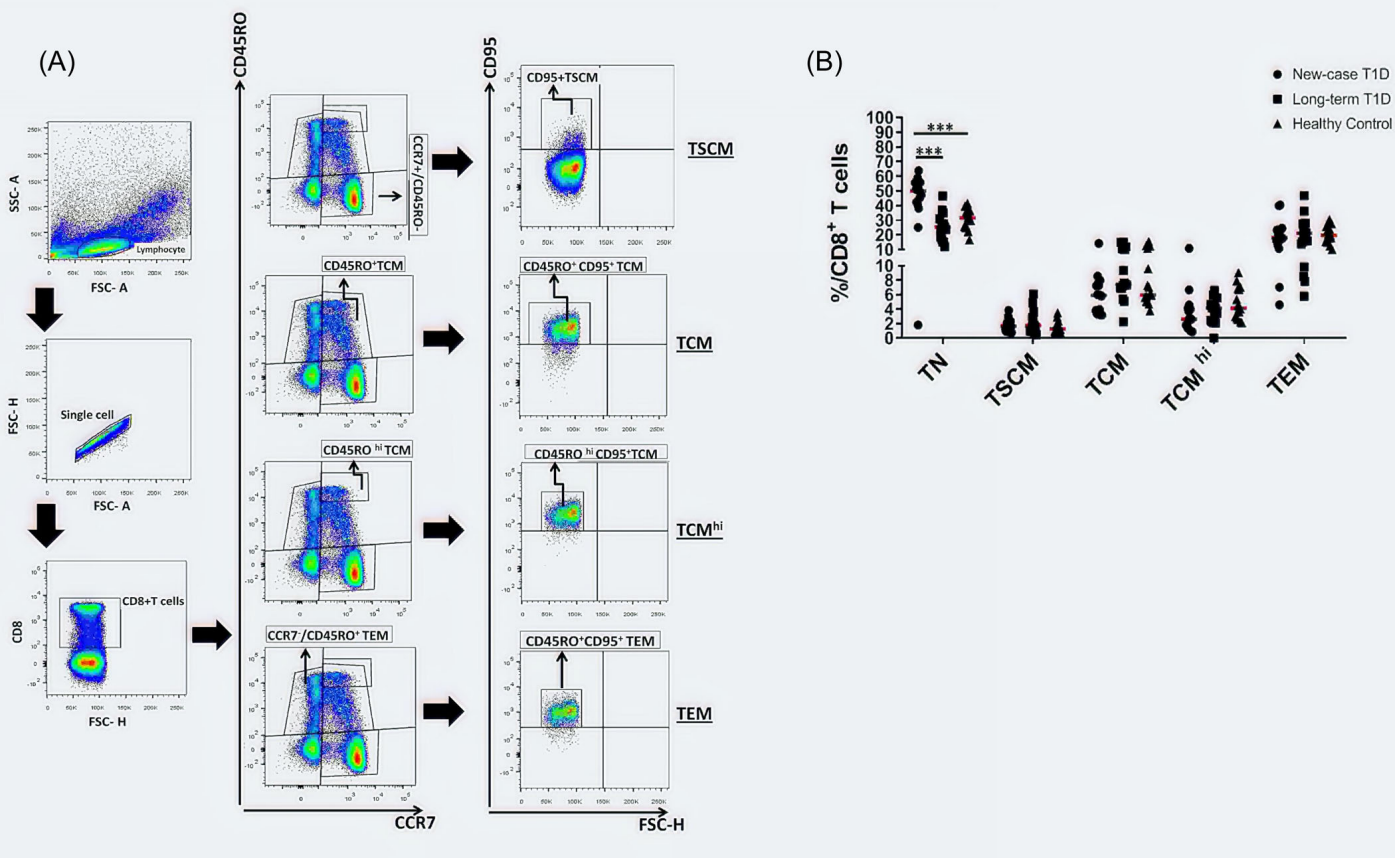
Changes in the percentages of CD8^+^T cell subsets in T1D patients. (A) The schematic diagram of gating of CD8^+^ T cell subsets in FCM. (B) The percentages of CD8^+^T cell subsets (TN, TSCM, TCM, TCM^hi^, and TEM) in new‐case T1D patients (*n* = 15), long‐term T1D patients (*n* = 15), and healthy controls (*n* = 15) are shown. Each symbol represents an individual. The means are represented by horizontal lines. *p < .05, **p < .01, ***p < .001, and ****p < .0001. Data analysis using the Kruskal–Wallis test followed by Bonferroni. TCM, T central memory.

The frequencies of CD8^+^ T cell populations were also analyzed in all study groups. The representative dot plots and the gating strategy used to detect CD8^+^ T cell subsets are shown in Figure 2A. The frequency of the CD8^+^CCR7^+^CD45RO^−^CD95^−^ T naive cells was higher in NC patients than in LT and healthy individuals (45.26 ± 15.26 vs. 25.37 ± 2.41, *p* = .0003 and 45.26 ± 15.26 vs. 30.46 ± 1.76, *p* = .0002, respectively, Figure 2B).

### 3.4 Frequency of PD‐1 expressing CD4^+^and CD8^+^ T cell subsets

**Figure 3:**
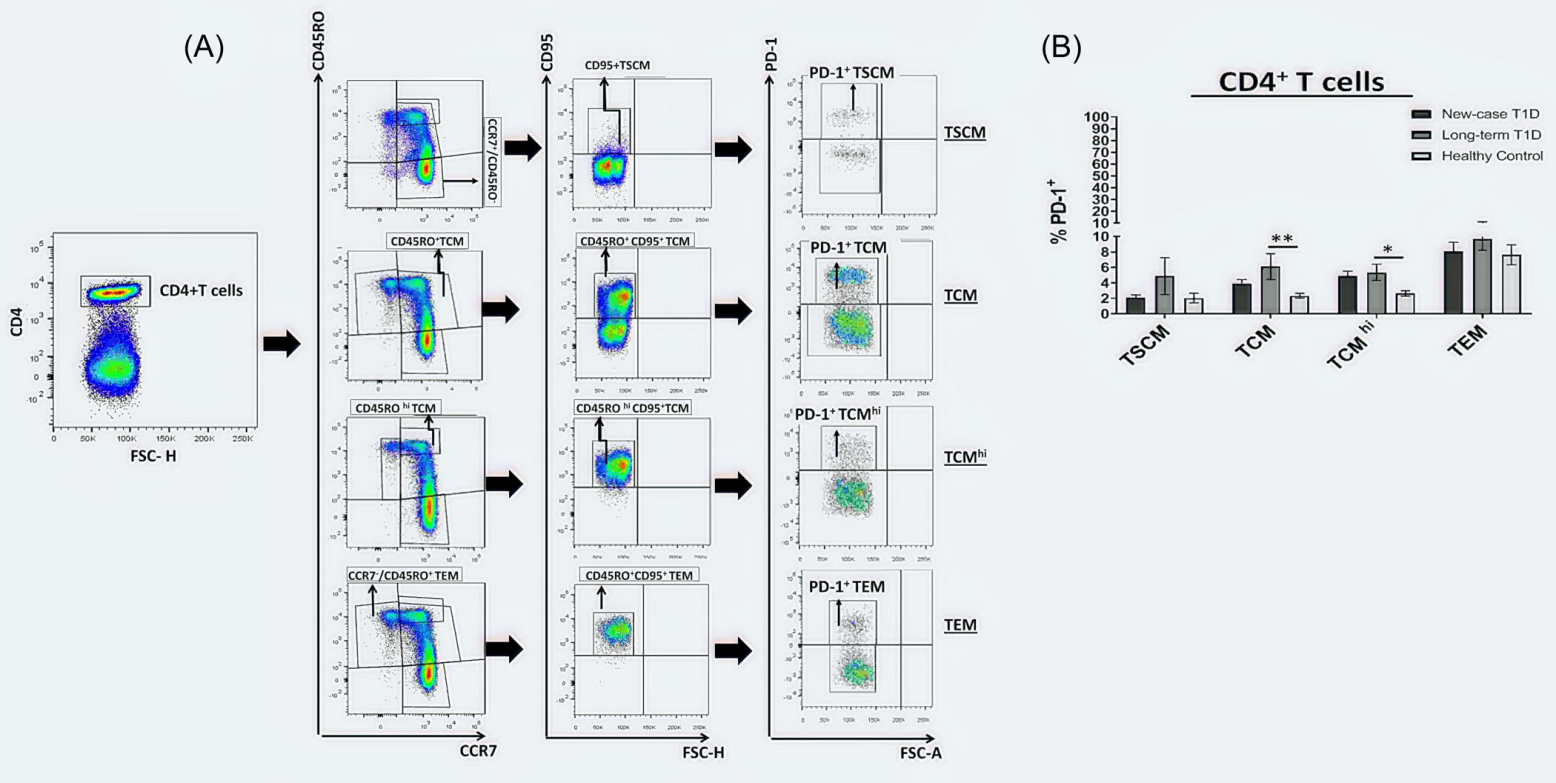
Inhibitory receptor expression on CD4^+^T cell subsets. (A) Representative dot plot of PD‐1 expression on CD4^+^ T cell subsets in T1D patients and healthy controls. (B) Surface PD‐1 expression level on CD4^+^ T cell subsets (TSCM, TCM, TCM^hi^, and TEM) were analyzed in new‐case T1D patients (*n* = 15), long‐term T1D patients (*n* = 15), and healthy controls (*n* = 15). Data are presented as mean ± SEM and analyzed by Kruskal–Wallis test followed by Bonferroni; *p < .05, **p < .01, ***p < .001, and ****p < .0001. TCM, T central memory.

**Figure 4:**
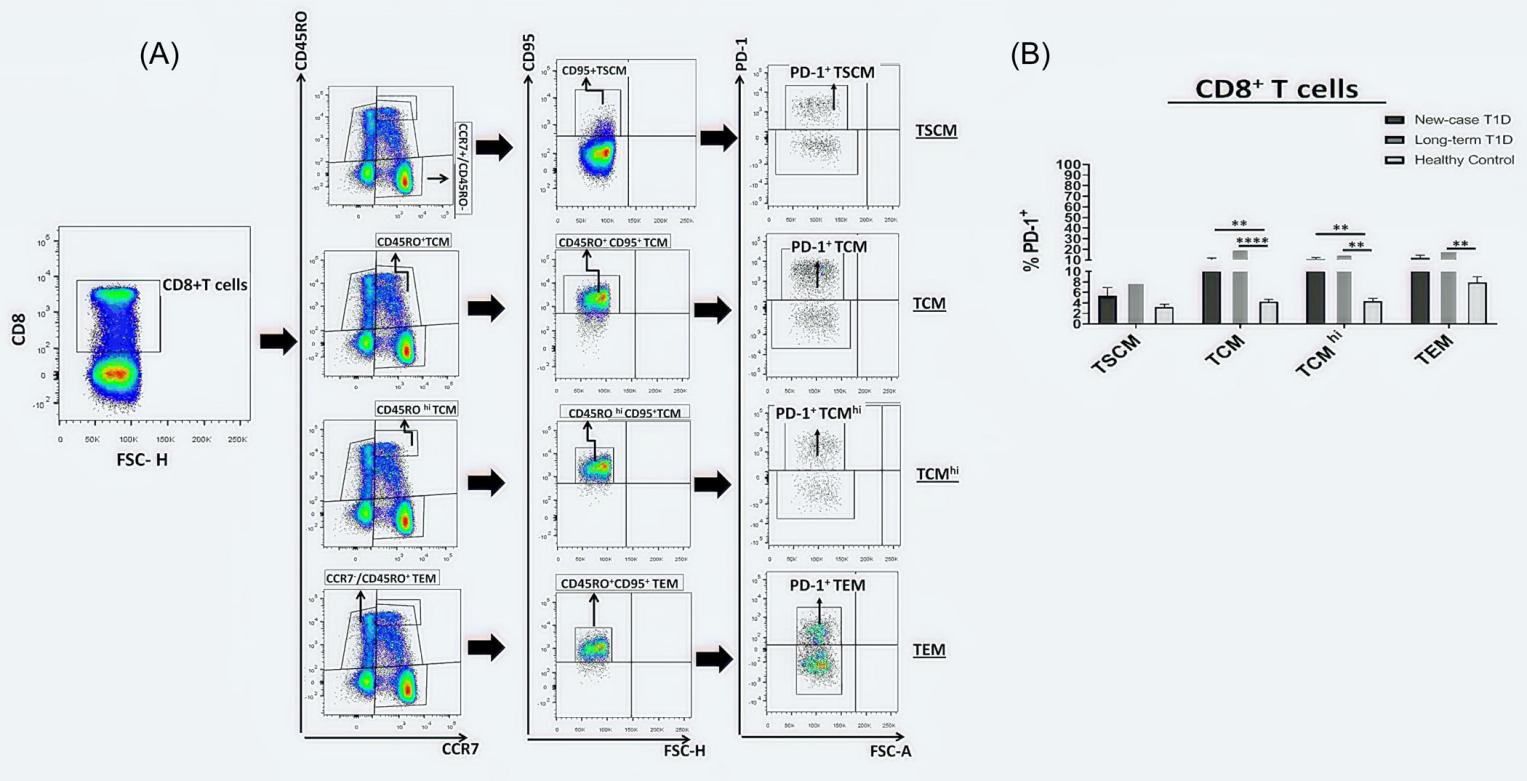
Inhibitory receptor expression on CD8^+^T cell subsets. (A) Representative dot plot of PD‐1 expression on CD8^+^ T cell subsets in T1D patients and healthy controls. (B) Surface PD‐1 expression level on CD8^+^ T cell subsets (TSCM, TCM, TCM^hi^, and TEM) were analyzed in new‐case T1D patients (*n* = 15), long‐term T1D patients (*n* = 15), and healthy controls (*n* = 15). Data are presented as mean ± SEM and analyzed by Kruskal–Wallis test followed by Bonferroni; *p < .05, **p < .01, ***p < .001, and ****p < .0001. TCM, T central memory.

We examined the frequency of PD‐1 expressing CD4^+^ or CD8^+^ T subsets in NC, LT and healthy groups, the representative dot plots of CD4^+^/CD8^+^ T cells, and the gating strategy used to detect PD‐1^+^CD4^+^ or PD‐1^+^CD8^+^ T cells are shown in Figures 3 and 4, respectively. In CD4^+^T cell subsets, the highest percentage of PD‐1 expression was observed on the CCR7^+^ CD45RO^+^ CD95^+^ TCM and CD4^+^ CCR7^+^ CD45RO^hi^ CD95^+^TCM^hi^ cells in LT patients as compared with healthy controls (6.09 ± 1.68 vs. 2.32 ± 0.3, *p* = .0037 and 5.35 ± 1.05 vs. 2.62 ± 0.35, *p* = .0145, respectively, Figure 3B). In CD8^+^ T cell subsets, the highest percentage of PD‐1 expression was observed on the CCR7^+^ CD45RO^+^ CD95^+^ TCM and CCR7^+^ CD45RO^hi^ CD95^+^TCM^hi^ in NC and LT patients as compared with controls (10.06 ± 1.84 vs. 4.27 ± 0.43, *p* = .0068 and 20.11 ± 5.14 vs. 4.27 ± 0.43, *p* < .0001; 10.59 ± 1.72 vs. 4.35 ± 0.53, *p* = .0012 and 15.71 ± 3.96 vs. 4.35 ± 0.53, *p* = .0012, respectively, Figure 4B). Furthermore, LT patients have a higher percentage of PD‐1 in their CD8^+^ CCR7^−^ CD45RO^+^ CD95^+^ TEM subsets as compared with healthy subjects (18.66 ± 3.12 vs. 7.97 ± 1.04, *p* = .0032, Figure 4B).

### 3.5 The relationship between different CD4^+^ and CD8^+^ T subsets

**Figure 5:**
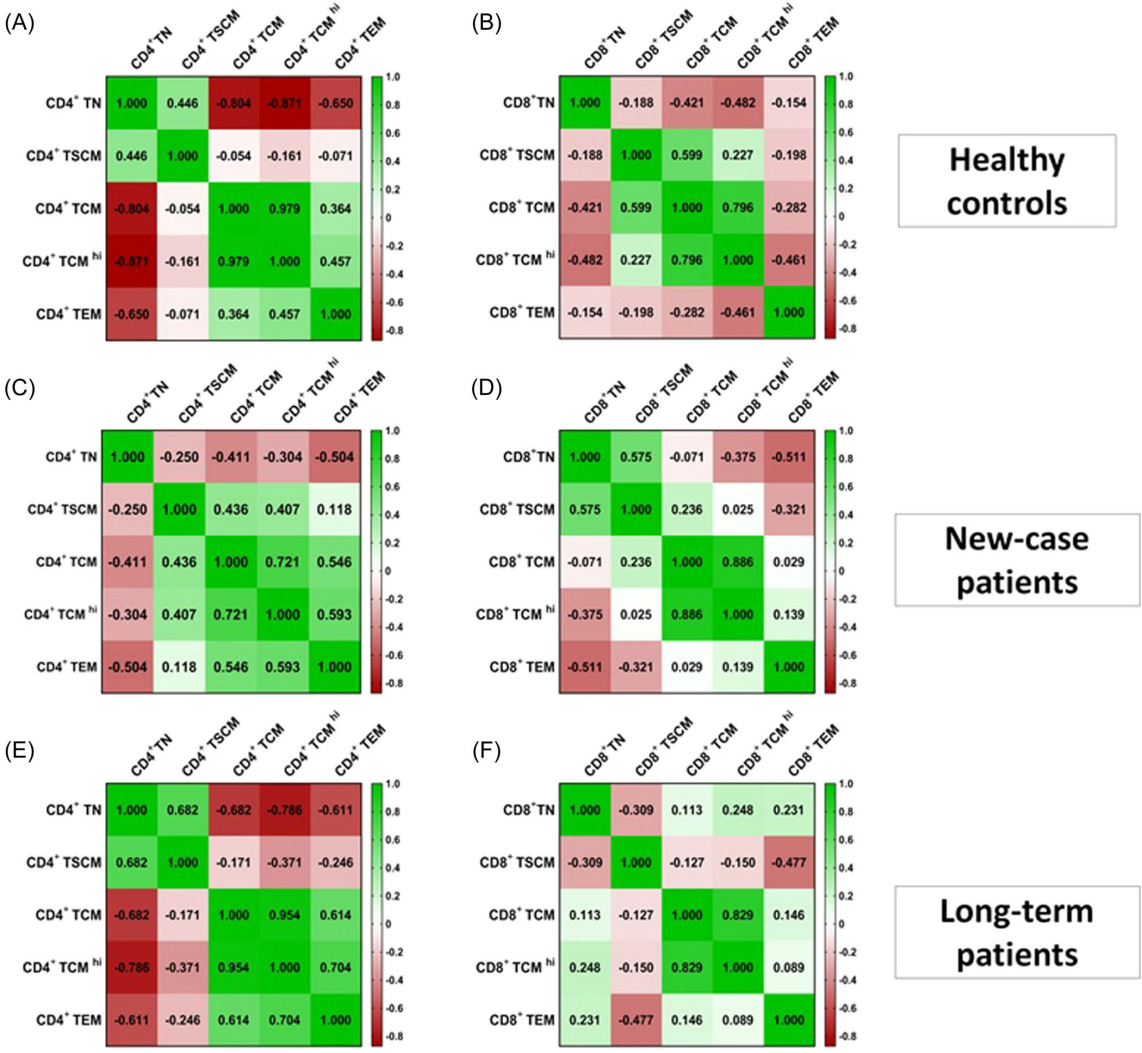
The correlation between CD4^+^and CD8^+^T cell subsets with each other in all study groups. (A) Heat map correlation of CD4^+^ T cell populations with each other in healthy controls. (B) Heat map correlation of CD8^+^ T cell populations with each other in healthy controls. (C) Heat map correlation of CD4^+^ T cell populations with each other in new‐case (NC) patients. (D) Heat map correlation of CD8^+^ T cell populations with each other in NC patients. (E) Heat map correlation of CD4^+^ T cell populations with each other in long‐term (LT) patients. (F) Heat map correlation of CD8^+^ T cell populations with each other in LT patients (green: positive correlation, red: negative correlation). The *p*‐value and *R* were determined according to Spearman's rank correlation test.

We analyzed the correlation between different CD4^+^ and CD8^+^ T subsets in all study groups. As shown in Figure 5A, the frequency of CD4^+^ naive T cells negatively correlated with frequencies of CD4^+^ TCM (*p* < .001; *r* = −0.804) and CD4^+^TCM^hi^ (*p* < .001; *r* = −0.871) in healthy controls. The percentage of CD4^+^ TCM positively correlated with CD4^+^TCM^hi^ cells in the healthy group (*p* < .001; *r* = 0.979, Figure 5A). Moreover, the frequency of CD8^+^ TCM was positively associated with CD8^+^TCM^hi^ (*p* < .001; *r* = 0.796) and CD8^+^ TSCM (*p* = .018; *r* = 0.599) cells in healthy subjects (Figure 5B). As shown in Figure 5C, significant positive correlations were found between the frequency of CD4^+^ TCM and CD4^+^TCM^hi^ (*p* = .002; *r* = 0.721) as well as CD4^+^ TEM (*p* = .035; *r* = 0.546) in NC individuals. The percentage of CD4^+^TCM^hi^ was positively correlated with CD4^+^ TEM (*p* = .020; *r* = 0.593). A positive correlation between CD8^+^ naive T and CD8^+^ TSCM (*p* = .025; *r* = 0.575) as well as CD8^+^ TCM and CD8^+^ TCM^hi^ (*p* < .0001; *r* = 0.886) were also found in the NC group (Figure 5D). As shown in Figure 5E, IN LT patients the percentage of CD4^+^ naive T cells was negatively correlated with CD4^+^ TCM (*p* = .005; *r* = −0.682), CD4^+^TCM^hi^ (*p* = .001; *r* = −0.786), CD4^+^ TEM (*p* = .016; *r* = −0.611) and was positively correlated with CD4^+^ TSCM (*p* = .005; *r* = 0.682). There were significant positive correlations between CD4^+^ TCM and CD4^+^TCM^hi^ (*p* < .0001; *r* = 0.954) and CD4^+^ TEM (*p* = .015; *r* = 0.614) in the LT group. The percentage of CD4^+^TCM^hi^ was positively correlated with CD4^+^ TEM (*p* = .003; *r* = 0.704) in LT patients. Furthermore, a positive correlation between CD8^+^ TCM and CD8^+^TCM^hi^ (*p* < .0001; *r* = 0.829) was also found in the LT group (Figure 5F).

## 4 DISCUSSION

The relevance of altered frequency and function of TSCMs in the wide spectrum of clinical conditions has received considerable attention recently. While TSCMs exhibit desirable effects in the context of infectious diseases and cancer immunotherapy, these cells may be detrimental in autoimmune diseases due to their stem cell‐like properties.^29^, ^35^, ^36^, ^37^, ^38^ Certainly, the long‐lived and self‐renewal characteristics of TSCMs may represent a reservoir of autoreactive lymphocytes with undesired and detrimental specificities responsible for autoimmune disease perpetuation.

In the current study, we evaluated the frequencies of CD4^+^ and CD8^+^ T subsets in patients with T1D. Our results demonstrated that the frequency of CD4^+^ TSCM was higher in NC patients than in LT cases and healthy controls. This finding is in line with a previous study which reported an increased percentage of CD4^+^ TSCM cells in patients with SLE and that isolated TSCM cells secreted IFN‐γ, TNF‐α, and IL‐2 and can differentiate into T follicular helper (Tfh) cells that provide B cell help to produce autoantibodies.^25^ Moreover, another study represented higher CD8^+^ TSCM cells in patients with AA and uveitis as well as higher CD4^+^ TSCM proportions in SLE patients.^24^ Interestingly, profound expansion of CD4^+^ TSCM is reported during acute HIV infection, which is related to disease progression.^39^ A higher frequency of CD4^+^ TSCM cells is shown to cause disease severity in rheumatoid arthritis (RA) patients.^40^ Indeed, these cells displayed the Th17 phenotype and were enriched in putative autoreactive specificities in RA patients compared to healthy donors.^40^ In patients with ITP, a higher frequency of CD4^+^TSCM has been associated with treatment efficacy and disease outcome.^27^ A recent study reported higher circulating autoreactive CD8^+^ TSCM cells specific for GAD65 and insulin in patients with T1D, which are generated in the presence of IL‐7 in vitro. They showed IL‐7 increases glucose uptake by overexpression of GLUT1 and oxidation of pyruvate in the mitochondria as well as upregulation of the glycolytic enzyme hexokinase 2, which are necessary for TSCM cells generation from naive precursors.^41^ Previous studies have shown that TSCMs are the least differentiated memory T cells and have introduced them as an intermediate stage between naive and conventional memory T cells.^30^, ^31^ Accordingly, these populations can differentiate into other memory and effector subsets.^16^, ^42^ Furthermore, they are antigen experienced and secrete cytokines such as TNF‐α, IFN‐γ, and IL‐2 following activation of T cells.^33^ Considering TSCMs' capacities to generate all memory and effector T cells, rapid proliferation and effector molecule generation after TCR stimulation, as well as a potential link between the IL‐7/IL‐7 receptor axis and the development of T‐cell responses toward β‐cells, we hypothesized that the increased frequency of TSCMs is associated with T1D progression.

We also showed the lower percentage of CD4^+^ TCM, TCM^hi^, and TEM cells as well as a higher frequency of CD8^+^ naive T cells in NC patients as compared with LT and healthy individuals. Further analysis of CD4^+^ and CD8^+^ T cell subsets in our study also revealed a negative association between the frequency of CD4^+^ naive T cells and the frequency of CD4^+^CM, CM^hi^, and EM T cells in LT patients and healthy controls. Moreover, our results showed positive correlations between the frequency of CD4^+^ and CD8^+^ memory T cells with each other in all 3 study groups. A previous study demonstrated that the percentages and absolute numbers of CD4^+^ CM cells were significantly reduced in recent‐onset diabetic patients compared with control subjects.^43^ Also, a lower frequency of CD4^+^ CM T cells was reported in acute progressive multifocal leukoencephalopathy (PML) that was preserved in patients with a fatal PML outcome.^44^ A significant increase in naive CD4^+^ and CD8^+^ T cells as well as a significant reduction in the frequency of CD4^+^ CM and EM T cells was found in patients with thyroid‐associated ophthalmopathy (TAO) as compared with controls.^45^ In addition, a higher percentage of CD4^+^ and CD8^+^ naive T cells was found in SLE patients.^25^ These data may represent dysregulated lymphocyte homeostasis, with favored generation or survival of naive T cells and increased sequestration of central and EM T cells in the inflamed tissues. Furthermore, in autoimmune disease settings, the constant presence of antigen and subsequent chronic nature of the ongoing immune responses may cause memory T cell exhaustion as well as reduced development of traditional memory response and consequently a reduction in their frequency.^46^, ^47^

Another important finding of our study was the higher expression of PD‐1 on CD4^+^/CD8^+^ TCM and TCM^hi^ in the PBMC of NC and LT patients. Previously, the higher expression level of PD‐1 was observed in CD4^+^ and CD8^+^ T cells in AA patients.^48^ In patients with SLE, higher expression level of PD‐1 was identified in CD4^+^ T cells, which is associated with IFN‐γ expression on CD3^+^ T cells.^49^ It is also reported that PD‐1 expression on tumor‐infiltrating T‐cells (TILs) correlates with tumor prognosis.^50^, ^51^ Moreover, increased PD‐1 expression in CD8^+^ TSCM in AA patients represented this subset as the least exhausted and self‐reactive T cell.^24^ Also, it has been reported that PD‐1 expression in healthy individuals correlates with differentiation into an EM phenotype.^24^, ^42^, ^52^ Therefore, it seems that increased expression of PD‐1 in CD4^+^/CD8^+^ TCM and TCM^hi^ of T1D patients might be associated with enhanced cytolytic effector activity and persistence of self‐reactive T cells.

In conclusion, we provide evidence for increased circulating CD4^+^ TSCM in T1D patients, highlighting the importance of this subset in the regulation of immune responses and the pathology of T1D by its ability to differentiate into effector and memory T cells. It would, therefore, be interesting to investigate the significance of TSCMs and their novel mechanistic insight in the progression of T1D and the prediction of disease outcome in a large longitudinal cohort of patients.

## AUTHOR CONTRIBUTIONS

**Pooriya Fazeli and Atefe Ghamar Talepoor**: Performed the experiments, analyzed and interpreted the data, and wrote the draft of the paper. **Zahra Faghih, Mohammad Reza Ataollahi, Mohammad Ali‐Hassanzadeh, and Hossein Moravej**: Conceived and designed the experiments and corrected the draft of the paper. **Nasser Gholijani and Kurosh Kalantar**: Conceived and designed the experiments, analyzed and interpreted the data, corrected the draft of the paper, and supervised the research.

## CONFLICT OF INTEREST

The authors declare no conflict of interest.

## Data Availability

Data sharing is not applicable—no new data is generated, or the article describes entirely theoretical research.
